# Best (but oft-forgotten) practices: the design, analysis, and interpretation of Mendelian randomization studies^1^

**DOI:** 10.3945/ajcn.115.118216

**Published:** 2016-03-09

**Authors:** Philip C Haycock, Stephen Burgess, Kaitlin H Wade, Jack Bowden, Caroline Relton, George Davey Smith

**Affiliations:** 2 Medical Research Council (MRC) Integrative Epidemiology Unit, University of Bristol, Bristol, United Kingdom; and; 3 Department of Public Health and Primary Care and; 4 MRC Biostatistics Unit, University of Cambridge, United Kingdom

**Keywords:** Mendelian randomization, causality, reverse causation, confounding, observational epidemiology, evidence synthesis

## Abstract

Mendelian randomization (MR) is an increasingly important tool for appraising causality in observational epidemiology. The technique exploits the principle that genotypes are not generally susceptible to reverse causation bias and confounding, reflecting their fixed nature and Mendel’s first and second laws of inheritance. The approach is, however, subject to important limitations and assumptions that, if unaddressed or compounded by poor study design, can lead to erroneous conclusions. Nevertheless, the advent of 2-sample approaches (in which exposure and outcome are measured in separate samples) and the increasing availability of open-access data from large consortia of genome-wide association studies and population biobanks mean that the approach is likely to become routine practice in evidence synthesis and causal inference research. In this article we provide an overview of the design, analysis, and interpretation of MR studies, with a special emphasis on assumptions and limitations. We also consider different analytic strategies for strengthening causal inference. Although impossible to prove causality with any single approach, MR is a highly cost-effective strategy for prioritizing intervention targets for disease prevention and for strengthening the evidence base for public health policy.

## INTRODUCTION

A major goal of applied epidemiology is to reduce the burden of disease in populations through interventions that target causal determinants of disease risk. However, because of the limitations of observational research, robust causal inference is usually not possible. Although observational studies, such as prospective cohort studies and case-control studies, can provide evidence with regard to disease etiology, limitations such as residual confounding, reverse causation bias, and measurement error severely constrain the ability to infer causality (1, 2). To get around such limitations, epidemiologists have traditionally relied on a set of empirical guidelines, often referred to as the Bradford Hill criteria (**Table 1**) (3). If these criteria are satisfied, the evidence for causality may be considered suitably robust to justify a randomized controlled trial (RCT)^5^ or public health policies for disease prevention. The criteria include temporality (exposure must precede disease), a dose-response relation, and specificity in the exposure-outcome relation (associations with specific disease outcomes, as opposed to a wide range of outcomes, are often viewed as more compatible with causality). In addition, although not formally part of the Bradford Hill criteria, associations should be independent of known confounders. However, even the most robust observational evidence, which typically comes from meta-analyses of prospective studies, will be susceptible to residual confounding (arising from measurement error and unknown confounders) and reverse causation bias (arising from preclinical stage of disease).

**TABLE 1:** Empirical criteria for appraising causality in epidemiology^1^

Criteria	Methods of appraising criteria
Temporality	Exposure precedes disease
Biological gradient	A dose-response relation between exposure and disease is present
Specificity	Greater specificity is often viewed as more compatible with causality—for example, when an exposure is associated with a single outcome as opposed to multiple outcomes
Independence	The exposure-disease association is independent of known confounders
Consistency/replication	A similar exposure-disease association is seen in independent studies
Strength	Weak associations are less likely to be causal
Plausibility	A plausible mechanism for the association exists
Coherence	Epidemiologic findings are compatible with laboratory evidence
Analogy	Effects exist for similar factors
Experiment	Interventions targeting the exposure are associated with reduced disease burden in the population

1 All of the criteria refer to Bradford Hill criteria (3), with the exception of “independence.”

The limitations of observational epidemiology are exemplified by research into vitamin E supplements, vitamin C, and HDL cholesterol and the risk of coronary artery disease (CAD). Although evidence from large meta-analyses of prospective studies supports an independent inverse association between HDL cholesterol and the risk of CAD (4), interventions designed to increase HDL cholesterol have not led to reductions in CAD incidence (5). Similarly, randomized trials of vitamin C and E supplementation did not indicate a decreased risk of cardiovascular disease (6), despite the evidence from prospective observational studies that suggested protective effects of these vitamins (7, 8).

Given that one of the primary rationales for observational studies is to inform intervention strategies for improving public health, such failures should be of major concern to researchers, funders, and public health policy makers. Moreover, given the expense and difficulty of designing RCTs and other prevention strategies, any approach that can strengthen causal inference, and so help prioritize intervention targets for disease prevention, is likely to save considerable resources. Mendelian randomization (MR) approaches have been developed with this context in mind and address many of the aforementioned limitations of observational epidemiology.

## MENDELIAN RANDOMIZATION

MR is a relatively new form of evidence synthesis and causal inference that is of growing importance in observational epidemiology (9). The approach can be viewed as an application of instrumental variable analysis, a technique originally developed in the field of econometrics (10), and exploits the principle that genotypes are not generally associated with confounders in the population and should be immune to reverse causation bias. These properties reflect both the fixed nature of genetic variants as well as Mendel’s first and second laws of inheritance. The key steps in the approach involve finding genetic polymorphisms to use as proxies, or “instruments,” for a target exposure and then testing the association of the genetic instrument with the outcome of interest (9, 11). An important advantage of the approach, which is of particular relevance to nutritional epidemiology, is that genotypes can be measured with very high accuracy and reflect long-term patterns of exposure. Thus, MR approaches are less susceptible to biases arising from measurement error.

For example, there is little doubt that circulating plasma concentrations of cholesterol are a causal determinant of risk for CAD. It is therefore widely accepted that genes involved in cholesterol pathways should be associated with the risk of CAD, as is observed empirically (12). Similarly, genes involved in smoking-related pathways should be associated with the risk of lung cancer. Formal approaches for estimating a causal effect include scaling the gene-outcome association to reflect a unit change in the exposure, which allows easier comparison with other estimates, such as those based on differences in the exposure from observational studies. Thus, if a genetic polymorphism is associated with a 0.5-SD change in cholesterol as well as a log OR for CAD of 0.1, this implies a log OR of 0.2 per 1-SD change in cholesterol (i.e., 0.1/0.5 = 0.2, known as a Wald ratio estimate).

Reflecting its growing importance, MR has been used to address the causal relevance of a wide range of modifiable exposures for risk of major noncommunicable diseases, including C-reactive protein (CRP) (13, 14), HDL cholesterol (15), uric acid (16) and triglycerides (17). Results from MR studies generally mirror those based on RCTs. For example, genetic proxies for HDL cholesterol are not generally associated with the risk of CAD (15, 18), consistent with the clinical trial evidence discussed above (5). In contrast, both genetic proxies for lower LDL cholesterol and interventions designed to lower LDL cholesterol are robustly associated with a lower risk of CAD (12, 19), providing particularly strong evidence for causality.

**FIGURE 1:**
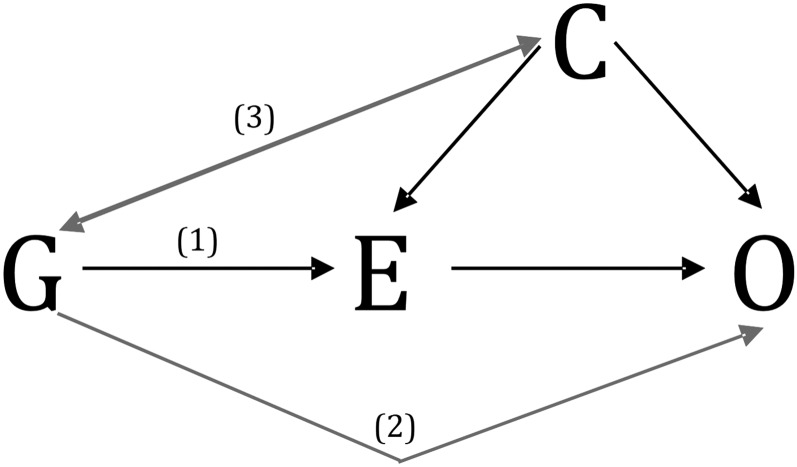
Basic principles of Mendelian randomization. The target exposure (E) is causally associated with the outcome (O) if the following conditions are held: *1*) the genetic variant (G) is associated with E; *2*) there is no association between G and O, except through E; and *3*) G is independent of any measured or unmeasured confounding factors (C). The gray lines indicate potential violations of Mendelian randomization assumptions and must be absent in order for G to be a valid instrumental variable. Reproduced from reference 23 with permission.

MR is, however, subject to important assumptions and limitations, which require careful consideration to avoid erroneous conclusions. The most important assumption pertains to the specificity of the gene-outcome association. Valid causal inference requires that the association between gene and outcome occurs exclusively through the hypothesized exposure. More formally, the genetic instrument *1*) must be associated with the exposure, *2*) should be independent of the outcome conditional on the exposure and confounders, and *3*) should not be associated with confounders of the exposure-outcome association (20–22) (**Figure 1**).

### Assumption 1

Valid causal inference in an MR study requires a true association between the genetic instrument and the exposure of interest. If this assumption is violated, lack of association between the genetic instrument and outcome may erroneously be interpreted as evidence against a causal association between exposure and outcome. Violation of this assumption is less likely when using biologically plausible genetic polymorphisms—for example, using variation in the gene for CRP as a proxy for CRP concentrations. Assumption 1 is the only 1 of the 3 assumptions that can be directly tested.

### Assumption 2

**FIGURE 2:**
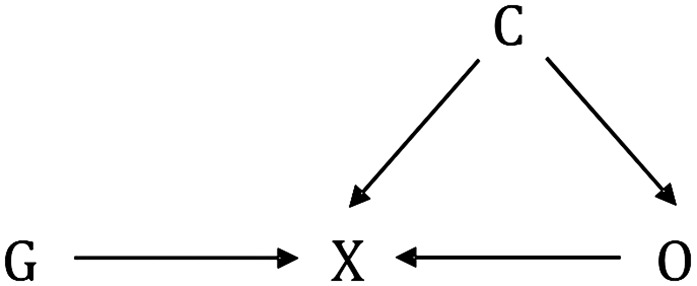
Collider bias. Conditioning on X, whether by design or analysis, induces a biased association between G and O, through C. C, confounder; G, genetic variant; O, outcome; X, exposure.

Valid causal inference requires that genetic instruments be independent of the outcome, conditional on the exposure and confounders of the exposure-outcome association. In other words, the effect of the genetic instrument on the outcome must be mediated exclusively by the exposure in question and there must be no direct effects. A direct effect is defined as a causal pathway between the genetic instrument and outcome that does not involve the hypothesized exposure. This assumption is described as “the exclusion restriction” in econometrics. Although it may be informative to check whether statistical adjustment for the exposure leads to attenuation of the gene-outcome relation (22), this adjustment can lead to collider bias (**Figure 2**) (24). Stratification of an MR analysis by exposure subgroups leads to a similar bias (see example below on gene-exposure interactions). The bias occurs when genotypes are not randomly distributed within the exposure subgroup strata, which may occur even if the genotype is randomly distributed in the population (24). The interpretation of such analyses is further undermined by potential measurement error in the exposure (25–28). Thus, complete attenuation of the genetic association with the outcome would not be expected even for a valid genetic instrument.

Direct effects can be introduced by a number of factors, including horizontal pleiotropy, linkage disequilibrium among gene loci (a known violation of Mendel’s second law of inheritance), and population stratification (9, 11, 29, 30). Horizontal pleiotropy occurs when a genetic variant affects multiple traits through separate pathways. Vertical pleiotropy, also known as mediation, in which a genetic variant is associated with multiple traits on the same pathway, is less problematic for MR studies. Linkage disequilibrium describes the correlation between genetic variants, typically for variants physically close together on the same chromosome. Population stratification occurs when subgroups within a sample have different genetic ancestries. These phenomena can introduce genetic confounding into an MR study.

### Assumption 3

The genetic instruments used in an MR study should not be associated with confounders (measured or unmeasured) of the exposure-outcome relation. Such associations can, however, occur by chance, especially when using weak instruments and small samples, a phenomenon known as weak instruments bias (31). Horizontal pleiotropy, linkage disequilibrium, and population stratification, as described above, can also induce associations between genetic instruments and confounders.

## LIMITATIONS OF MR STUDIES

In addition to the aforementioned assumptions and potential violations, MR is susceptible to other important practical and theoretical limitations. These include the difficulty of finding genetic instruments for hypothesized exposures, low statistical power, “winner’s curse,” limited biological understanding of gene-exposure associations, trait heterogeneity, canalization, and limitations of estimating associations for binary outcomes.

### Difficulty of finding genetic instruments

Finding genetic polymorphisms to use as genetic instruments in an MR study is becoming ever more feasible with the growing proliferation of genome-wide association studies (GWASs). In a GWAS, the association of hundreds of thousands to millions of genetic variants, typically single nucleotide polymorphisms (SNPs), are tested for an association with a trait of interest. Finding suitable genetic instruments can, however, be challenging. For example, in our MR studies of lung cancer, we found that only 57% of putative risk factors were “instrumentable” (i.e., were subject to known associations with SNPs). To date, most large-scale GWASs have been conducted on comparatively easy or inexpensive traits to measure.

### Low power

Because genetic polymorphisms typically explain only a small fraction of the total variance in traits, MR studies require very large sample sizes for sufficient statistical power. For example, when a genetic instrument explains 1% of the variation in a trait, which is not unusual, ∼9500 cases and equal number of controls will be required for 80% power to detect an OR of 1.5 per SD change in the exposure, assuming a *P* value threshold of 0.05 for significance (32). For individual polymorphisms, the variance explained will typically be <1%, which is why it is usually advisable to combine multiple polymorphisms into a single allele score, so as to maximize the explanatory power of the instrument. Strong multigene instruments are, however, the exception rather than the rule. For example, although 97 genetic loci have been implicated in BMI by GWASs, these loci together account for just 2.7% of the total variance in BMI (33).

### Winner’s curse

When GWASs report evidence of association for a trait at a specific genomic region, involving multiple, sometimes hundreds, of SNPs, they typically select the SNP with the smallest *P* value as the lead SNP and do not report the associations for other significant SNPs. This practice generally leads to overestimation of the SNP-trait effect, also known as the winner’s curse or Beavis effect (34). The overestimation occurs because of chance correlation between SNPs and confounders in the GWAS discovery stage. If the GWAS discovery and MR studies are independent, the winner’s curse will not affect the power or size of a causal hypothesis test, but it will bias MR estimates toward the null. To illustrate this, consider the Wald ratio, a common approach for deriving causal estimates from summary data with a single SNP. The Wald ratio is the coefficient of the SNP-outcome association divided by the coefficient of the SNP-exposure association. Thus, overestimation in the denominator, e.g., due to winner's curse, will result in an underestimation of the ratio but only when both samples are independent. When the discovery and MR analysis samples are the same, both the numerator and denominator will be overestimated.

### Poor biological understanding

**FIGURE 3:**
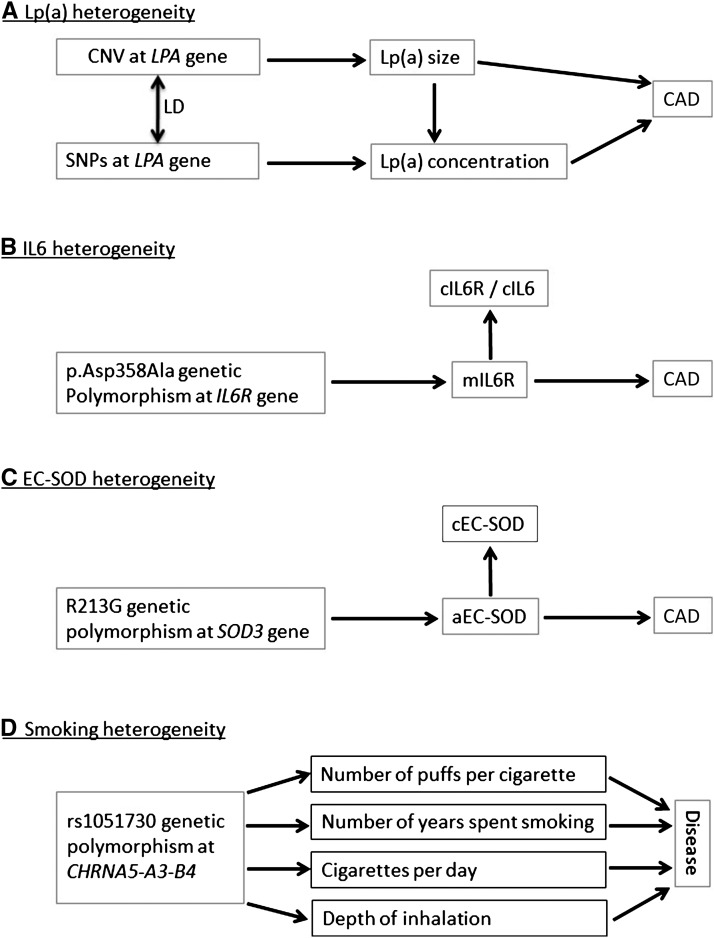
Trait heterogeneity and causal inference in Mendelian randomization studies. The figure shows how trait heterogeneity can undermine causal inference in Mendelian randomization studies. (A) The association of Lp(a) concentration with the risk of CAD is confounded by Lp(a) size; (B) the associations of cIL6 and cIL6R concentrations with risk of CAD are confounded by mIL6R; (C) the association between cEC-SOD and CAD is confounded by aEC-SOD; and (D) the association between rs1051730 and disease is likely to be mediated by multiple dimensions of smoking behavior. aEC-SOD, arterial/endothelial extracellular superoxide dismutase; cEC-SOD, circulating extracellular superoxide dismutase; CAD, coronary artery disease; *CHRNA5-A3-B4*, cholinergic receptor nicotinic α 5 subunit - α 3 subunit - β 4 subunit, nicotinic receptor gene cluster; cIL6, circulating IL-6; cIL6R, circulating IL-6 receptor; CNV, copy number variant; EC-SOD, extracellular superoxide dismutase; LD, linkage disequilibrium; Lp(a), lipoprotein(a); *LPA*, apolipoprotein(a) gene; mIL6R, membrane-bound IL-6 receptor; SNP, single nucleotide polymorphism; *SOD3*, superoxide dismutase 3 gene.

Although thousands of SNP-trait associations have been discovered by GWASs, little is typically understood about the underlying biology or mechanisms of association. This limitation can sometimes lead to counterintuitive results. For example, oxidative processes are strongly implicated in atherosclerosis, the major driver of CAD, and cardioprotective effects have therefore been hypothesized for antioxidants. However, contrary to this expectation, the R213G genetic polymorphism in the gene for extracellular superoxide dismutase (EC-SOD), an extracellular scavenger of superoxide anions, is associated with higher circulating concentrations of EC-SOD as well as a higher risk of CAD (35). However, the higher concentration in blood is thought to result from depletion of EC-SOD in the arterial wall. Thus, the R213G genetic polymorphism should be associated with a reduced concentration of EC-SOD at the site of atherosclerosis, which is of more direct biological relevance to CAD. Without this additional biological knowledge, the positive association between circulating EC-SOD and CAD risk could naively be interpreted as evidence supporting a cardio-deleterious role for antioxidants (**Figure 3**).

A similarly counterintuitive result is seen in MR studies of the IL-6 receptor (IL-6R) and CAD (36, 37). The p.Asp358Ala genetic polymorphism at the *IL6R* gene is associated with higher circulating concentrations of IL-6R and IL-6 and reduced odds of CAD, contrary to the expectation from observational studies in which a higher circulating concentration of IL-6 is associated with higher risk. The paradox can be explained by reduced membrane-bound IL-6R, leading to increased circulating concentrations of IL-6R and IL-6 and reduced IL-6 cell signaling. In other words, the higher circulating concentrations of IL-6 in carriers of p.Asp358Ala do not stem from increased production of the protein but rather from reduced cellular binding of IL-6. As with the EC-SOD example, biological knowledge about the IL-6 pathway prevented erroneous causal inference. Unfortunately, such detailed biological knowledge is unlikely to apply to the majority of known gene-trait associations.

### Trait heterogeneity

Genetic polymorphisms are sometimes associated with multiple aspects or dimensions of a single trait (Figure 3). Such trait heterogeneity does not preclude causal inference but it does undermine the ability to infer causality for particular dimensions of heterogeneous traits. For example, a copy number variant at exons 4 and 5 of the apolipoprotein(a) gene (*LPA*) affects both the size and expression levels of the protein product, apolipoprotein(a) [apo(a)]. Posttranslationally, apo(a) is covalently bound to LDL to form lipoprotein(a) [Lp(a)], a well-established risk factor for CAD. Observationally, smaller protein size and higher concentrations of Lp(a) are associated with higher CAD risk (38, 39). MR studies suggest that the associations reflect causality, because genetic polymorphisms associated with Lp(a) concentration are associated with CAD (40, 41). However, the association between Lp(a) concentration and CAD is potentially confounded by apo(a) size, because the size-determining copy number variant could affect CAD independently of its effect on Lp(a) concentration (42–47). Thus, although there is little doubt that a causal association exists between the Lp(a) particle and CAD, ascertaining the particular dimension of Lp(a) driving the association with CAD is undermined by Lp(a) heterogeneity (Figure 3).

There are parallels between the above example for Lp(a) and the problematic interpretation of the findings for IL-6R and EC-SOD, described above, which were also subject to confounding by different dimensions of the same trait (Figure 3). For example, in the case of IL-6R, the association between circulating IL-6R and CAD was confounded by reduced IL-6 cell signaling. Only biological knowledge was able to resolve the particular dimension of the IL-6 pathway that was causally relevant to increased CAD risk.

Trait heterogeneity may also limit MR studies of smoking, as shown by the example of the nicotinic receptor gene cluster on chromosome 15 [cholinergic receptor nicotinic α 5 subunit - α 3 subunit - β 4 subunit (*CHRNA5-A3-B4*)]. Each copy of the minor allele of rs1051730 (or equivalently rs16969968), which resides within the gene cluster, is associated with 1 extra cigarette smoked per day. The latter behavior is, however, only one among several dimensions of smoking behavior that affect tobacco exposure (48). Smokers may vary in their depth of inhalation, the number of puffs they take per cigarette, how much of the cigarette they smoke, the type of cigarette they smoke, and number of years spent smoking, all of which will affect total lifetime exposure to tobacco (Figure 3). Thus, it is unlikely that the association between the nicotinic receptor and tobacco exposure is mediated exclusively by cigarettes smoked per day. Heterogeneity in smoking behaviors may explain why rs1051730 and rs16969968 are more strongly associated with cotinine (an objective biomarker of tobacco exposure) than with daily cigarette consumption (49). Although this heterogeneity in behavior does not invalidate the use of *CHRNA5-A3-B4* as a genetic instrument for smoking, it does undermine its use for estimating causal effects for particular dimensions of smoking behavior (48). In other words, we may still be able to infer that a causal association exists but be unable to infer the precise magnitude of the causal effect.

### Canalization

During development, compensatory processes may be generated that counter the phenotypic impact of the genetic variant being used as an instrument for an exposure of interest (11). Theoretically, one could expect such developmental buffering to result in attenuated or null associations between the genetic instrument and outcome. Canalization is likely to be less problematic for exposures adopted after fetal development, such as behavioral traits like smoking, and more of a problem for severe protein-coding mutations expressed during fetal development. No general approaches are available for appraising or avoiding canalization.

### Estimating associations for binary outcomes

If the outcome is binary (e.g., a disease outcome) and particularly if the genetic associations with the outcome are estimated in a case-control setting, then estimates of the MR effect of exposure on outcome may only be approximate (50). If exposure measurements are obtained after disease diagnosis in a case-control setting, then the genetic associations with the exposure could be biased by reverse causation unless the gene-exposure association is derived from controls only (21). Estimates will be less biased in a nested case-control study where measurements are obtained at baseline, but participants should still be weighted so that the case-control sample better represents the target population (51). In addition, because of the phenomenon of “noncollapsibility,” ORs have limited generalizability (52–54). Noncollapsibility, which does not apply to other etiologic estimates such as HRs or risk differences, derives from the mathematics of how an OR is calculated (54). In practice, noncollapsibility means that ORs cannot be used to predict the precise impact of an intervention on specific subgroups in a population and can only be used to predict the population-averaged causal effect (53, 54). The above considerations do not affect the ability of an MR study to draw unbiased conclusions about whether a target exposure has a causal effect on an outcome (53), but they do affect the magnitude of the estimated effect (54).

## CONSTRUCTING A GENETIC INSTRUMENT

The most important step in an MR study is finding a genetic instrument to use as a proxy for the target exposure. In practice, most genetic instruments used to date have been based on SNPs, but in principle, any kind of genetic polymorphism could be used, including copy number variants (41). In the first step of constructing a genetic instrument it is important to find genetic polymorphisms robustly associated with the exposure of interest, for which there are 2 broad approaches. One approach is to use polymorphisms with proven or plausible biological effects on the target exposure—for example, using SNPs at the *CRP*, alcohol dehydrogenase 1B (*ADH1B*), and *IL6R* genes as instruments for CRP, alcohol, and IL-6R, respectively. Results based on such instruments can be particularly compelling because they may be less susceptible to pleiotropy and more likely to reflect the target exposure. The second general approach exploits the increasing availability of results from GWASs. Hundreds of such studies have been conducted to date, and their findings are curated by the National Human Genome Research Institute’s GWAS catalog. Currently there are >10,000 SNP-trait associations curated by the catalog, corresponding to ∼1400 unique phenotypes. In principle, SNPs curated by the catalog can be used to construct genetic instruments for exposures of interest.

An important caveat is that little of the underlying mechanisms of the associations curated by the catalog will typically be well understood, which increases the potential for pleiotropy and counterintuitive results (as discussed above). In addition, many of the SNPs curated by the catalog have not been replicated in independent samples, increasing the potential for false positives as well as winner’s curse bias. On the other hand, the number of SNPs available for instrumentation is likely to be far greater when relying on GWASs, as opposed to hypothesis-driven approaches. When multiple SNPs are available, these can be combined into a single genetic instrument to increase the statistical power of an MR study.

## DIFFERENT DESIGN STRATEGIES FOR MR

In a broad sense, any approach that uses genetic information to make inferences about the causal relation between traits could be considered part of the MR family. As a result, there are many different design strategies for MR (**Table 2**). Some of the strategies provide estimates of the magnitude of the causal effect, whereas others only provide evidence on whether a causal association exists. The strategies considered below can be applied equally to continuous and binary (e.g., disease) outcomes, although, as noted above, estimation in the binary setting may only be approximate.

**TABLE 2:** Different design strategies for MR^1^

Study design	Test	Comments
G-X + G-Y	Implies X→Y	No estimation of magnitude of causal effect
One-sample MR	Various hypotheses	Requires individual-level data; lower power; MR estimates are biased toward the confounded observational association by weak instruments
Two-sample MR	Various hypotheses	Individual-level or summary data; greater power (due to greater potential sample sizes); MR estimates are biased toward the null by weak instruments
Bidirectional MR	X→Y and Y→X	Assesses causation in both directions
Two-step MR	X→M→Y	Tests mediation in a causal pathway
G×E	X→Y (relation is dependent on environment variable)	Able to detect direct effects (a violation of assumption 2 of MR)

1 G×E, gene-environment interaction; G-X, SNP-exposure association; G-Y, SNP-outcome association, M, mediator; MR, Mendelian randomization; SNP, single nucleotide polymorphism; X, hypothesized exposure; Y, outcome variable of interest.

### Gene-exposure plus gene-outcome association

In the simplest study design, the existence of a gene-exposure and a gene-outcome association implies a causal effect of exposure on outcome. For example, in one study, genetic polymorphisms at the *LPA* gene were associated with both Lp(a) concentration and the risk of CAD (40). Although the magnitude of the causal effect of Lp(a) on CAD was not formally estimated, these findings indicate that the positive association between Lp(a) concentration and CAD risk (39) reflects causality. The assumptions behind this approach can be viewed as less stringent in comparison with techniques that attempt to estimate the magnitude of the causal association, described below.

### One-sample MR with individual participant data

One-sample MR is the standard implementation of MR in a single data set with data on the SNPs, exposure, and outcome for all participants. With individual participant data, the causal effect of the exposure on the outcome can be estimated by using 2-stage least-squares (2SLS) regression, a method originally developed in the field of econometrics (55). In the first stage, the exposure of interest is regressed on the genetic instrument, which can either be a single SNP, multiple SNPs, or an allele score based on multiple SNPs (e.g., the sum of the number of exposure-increasing alleles). The predicted values of the exposure are taken from the first-stage regression model. In the second stage, the outcome of interest is regressed over the predicted values of the exposure by using either linear or logistic regression, depending on whether the outcome is a continuous or binary variable. The β-coefficient from the second stage can be interpreted as the change in the outcome (for logistic regression, the log OR for disease) per unit increase in the exposure due to the genetic instrument. When using an allele score, it is typical for the SNPs to be weighted according to the size of the gene-exposure effect from an independent study. When implementing 2SLS, it is important that the samples used for discovering the genetic instrument or instruments are independent of the samples used for the MR analysis. Overlap between discovery and analysis samples compounds the effect of weak instruments, biasing causal estimates toward the confounded observational association. 2SLS can be implemented by using standard statistical software, including “ivregress” in STATA (StataCorp) and “ivpack” in R (R Foundation).

### Summary data

Summary data are summarized genetic associations with the exposure and outcome (usually in the form of β-coefficients and SEs) often provided by consortia when sharing individual-level data are impractical. A common approach for deriving causal estimates from summary data with a single SNP is the Wald ratio, in which the coefficient of the SNP-outcome association is divided by the coefficient of the SNP-exposure association. If the outcome is a binary disease trait, the Wald ratio can be interpreted as the log OR for disease per unit increase in the exposure due to the SNP. This gives the same estimate as the 2SLS method with a single SNP. The SE of the Wald ratio can be estimated as the SE of the gene-outcome association divided by the coefficient of the gene-exposure association, but this does not take into account uncertainty in the latter. Alternatively, the SE of the Wald ratio can be approximated by the delta method, which makes allowance for uncertainty in the gene-exposure and gene-outcome relations (56).

A number of approaches exist for combining summary data across multiple SNPs. A common approach is to use weighted linear regression, in which the coefficient of the gene-outcome association is regressed on the coefficient of the gene-exposure association, with weights derived from the inverse variance of the gene-outcome association, and with the intercept constrained to zero. The slope from this model can be interpreted as the MR estimate of the effect of the exposure on the outcome. The slope of the relation between the gene-outcome and gene-exposure associations can also be estimated by maximum likelihood (57). Alternatively, Wald ratios can be estimated for each SNP separately and combined by fixed- or random-effects meta-analysis. Methods based on summary data generally require that the SNPs be completely independent or that the correlation between SNPs be taken into account—for example, through a variance-covariance matrix of the SNPs based on 1000-genomes data (58). The effect estimates from all of these approaches should be equivalent to the effect estimated by an allele score in 2SLS when sample sizes are large and SNPs are completely independent.

### Two-sample MR

Two-sample approaches are a novel extension to 2SLS that greatly increase the scope of MR, because they allow for greater sample sizes and thus greater statistical power. Contrary to a 2SLS approach, in which the gene-exposure and gene-outcome relations are estimated in the same sample, 2-sample approaches derive the estimates from separate samples (e.g., separate GWASs of exposure and outcome) (57, 58). The major assumptions of the approach are that the gene-exposure and gene-outcome associations should be estimated in nonoverlapping samples and should be representative of the same population, practically defined as being of similar age and sex distribution and the same ethnic group. When the latter assumption is violated, the approach may still provide evidence on whether a causal association exists but not necessarily on the precise magnitude of the causal effect. In a 1-sample setting, bias of causal estimates due to weak instruments is toward the confounded observational association, whereas in a 2-sample setting, bias is toward the null. Another advantage of the approach is that access to individual-level data is not required and causal estimates can be derived from summary data alone (57). An important consideration is that the gene-exposure and gene-outcome associations should be coded relative to the same effect allele. Harmonization of the effect alleles is usually straightforward but may be problematic for palindromic SNPs (i.e., G/C and A/T SNPs), which look the same on both DNA strands. If the reference strand is unknown in one or both samples, it can be inferred from the frequency of the effect allele.

Two-sample approaches exploit the growing availability of summary data from large consortia of GWASs, such as the GIANT (Genetic Investigation of ANthropometric Traits) consortium, the Global Lipids Genetics Consortium, the International Consortium for Blood Pressure, and the CARDIoGRAM (Coronary ARtery DIsease Genome wide Replication and Meta-analysis) consortium. **Table 3** provides a nonexhaustive list of publicly available summary data. A list of available studies can also be found on the website of the psychiatric genomics consortium (59) and in Burgess et al. (57).

**TABLE 3:** Publicly available GWAS summary data^1^

Trait	Consortium
Alzheimer disease	International Genomics of Alzheimer's Project
Anorexia nervosa	Genetic Consortium for Anorexia Nervosa
Anthropometric traits	Early Growth Genetics Consortium
Anthropometric traits	Genetic Investigation of ANthropometric Traits (GIANT)
Autism	Psychiatric Genomics Consortium
Bipolar disorder	Psychiatric Genomics Consortium
Blood pressure	International Consortium for Blood Pressure
Coronary artery disease	Coronary ARtery DIsease Genome wide Replication and Meta-analysis (CARDIoGRAM)
Crohn disease	International Inflammatory Bowel Disease Genetics Consortium
Education	Social Science Genetics Association Consortium
Glycemic traits	Meta-Analyses of Glucose and Insulin-related traits Consortium (MAGIC)
Lipids	Global Lipids Genetics Consortium
Major depressive disorder	Psychiatric Genomics Consortium
Osteoporosis	GEnetic Factors for OSteoporosis Consortium (GEFOS)
Schizophrenia	Psychiatric Genomics Consortium
Smoking	Tobacco and Genetics Consortium
Type 2 diabetes	DIAbetes Genetics Replication And Meta-analysis (DIAGRAM)
Ulcerative colitis	International Inflammatory Bowel Disease Genetics Consortium

1 GWAS, genome-wide association study.

### Bidirectional MR

**FIGURE 4:**
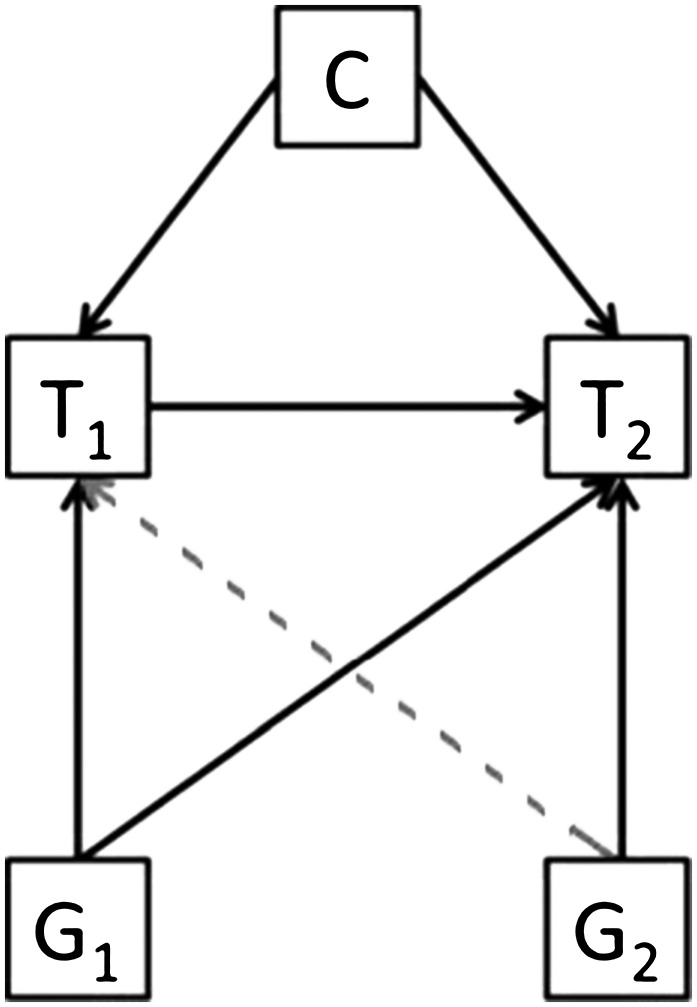
Bidirectional Mendelian randomization. If a trait (T_1_) is causally associated with another (T_2_), then the genetic variant associated with T_1_ (G_1_) will be associated with both T_1_ and T_2_. However, the reverse (gray dashed line) will not be true and the genetic variant associated with T_2_ (G_2_) will not be associated with T_1_ (unless the relation is truly bidirectional). Reproduced from reference 23 with permission.

In bidirectional, or reciprocal, MR, instruments for both exposure and outcome are used to assess the causal association in both directions (**Figure 4**). For example, Timpson et al. (60) used this approach to assess the direction of causation between circulating CRP concentrations (instrumented by the rs3091244 SNP in the *CRP* gene) and BMI [instrumented by the rs9939609 SNP in the fat mass and obesity associated (FTO) gene]. Within this context, the authors showed that CRP is likely to be a marker of increased adiposity, rather than the reverse.

This approach allows further dissection of the directional association between an exposure and outcome and can be extended to networks of correlated phenotypes (60–62). However, a potential limitation is that the approach assumes that the causal association occurs in one direction, such that the impact of feedback loops between exposure and outcome cannot be addressed.

### Two-step MR

**FIGURE 5:**
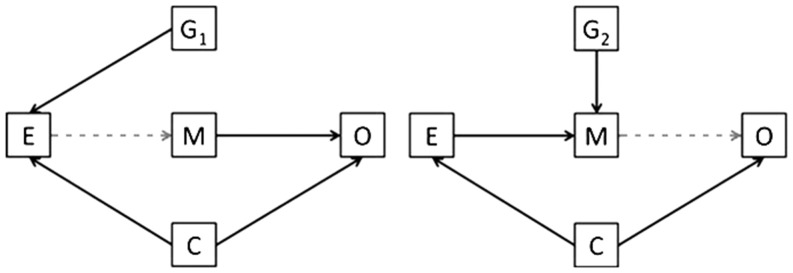
Two-step Mendelian randomization. In step 1 (left diagram), an SNP (G_1_), independent of any confounding factors (C), is used as a genetic proxy for an exposure (E) to test the impact of E on a hypothesized mediator (M) of an E-outcome (O) association. G_1_ will influence M only if E is causally related to M (gray dashed line). In the second step (right diagram), another independent SNP (G_2_) is similarly used as a proxy for M to assess the causal association between M and O (gray dashed line). Reproduced from reference 23 with permission. SNP, single nucleotide polymorphism.

Two-step MR, not to be confused with 2-sample MR, uses genetic instruments to assess mediation in a potentially causal pathway (**Figure 5**) (11, 63–65). For example, it may be desirable to know whether blood pressure mediates the association between BMI and CAD. In the first step, genetic instruments for BMI are used to assess the causal association between BMI and blood pressure. In the second step, genetic instruments for blood pressure are used to assess the causal effect of blood pressure on CAD risk. Evidence of association in both steps implies some degree of mediation of the association between BMI and CAD by blood pressure. The magnitude of the direct and indirect effects of BMI on CAD can also be estimated (64, 65); however, this requires the assumptions of linearity and homogeneity in both the exposure-mediator and exposure-outcome associations (65). In addition, there should be no interaction between exposure and mediator (i.e., the association of the exposure with the outcome should not vary by values of the mediator and vice versa) (65).

### Gene-exposure interactions

The existence of a gene-exposure interaction implies that the association between gene and outcome varies by exposure status. This can be exploited by MR studies to distinguish between a direct (pleiotropic) effect of the gene on the outcome and a causal effect via the exposure. For example, the *CHRNA5-A3-B4* gene cluster is associated with heaviness of smoking and lung cancer (66). Assuming satisfaction of MR assumptions, the *CHRNA5-A3-B4* genetic cluster should not be associated with lung cancer in never-smokers. Such an association would indicate the presence of an alternative pathway from *CHRNA5-A3-B4* to lung cancer that does not involve smoking (i.e., a direct effect). Gene-exposure interactions have been used to assess the causal relevance of alcohol for risk of upper digestive cancer and systolic blood pressure by using genetic polymorphisms at the *ADH1B* and aldehyde dehydrogenase 2 (*ALDH2*) genes, which code for alcohol dehydrogenase 1B and aldehyde dehydrogenase 2, respectively (67, 68). These genetic instruments for alcohol consumption were associated with cancer and blood pressure in men but not women. Given that all drinkers in the latter study were male and all nondrinkers female, the results are consistent with the assumption that the instruments work exclusively through alcohol metabolism. A potential limitation of using gene-exposure interactions for assessing causal hypotheses is that stratification into exposure subgroups can induce noncausal associations between genotype and outcome as a result of collider bias. For example, in an MR study of whether milk is causally associated with the risk of type 2 diabetes (69), genotypes at the gene for lactase, a known determinant of milk consumption, were associated with increased risk of type 2 diabetes but only in non–milk consumers, which the authors attributed to collider bias (Figure 2).

## PRACTICAL STRATEGIES FOR ENHANCING CAUSAL INFERENCE

Genetic confounding (e.g., due to horizontal pleiotropy) and limited power are the most important limitations typically faced in an MR study. In this section we consider a number of practical strategies for dealing with these limitations and for enhancing causal inference (summarized in **Table 4**).

**TABLE 4:** Practical strategies for enhancing causal inference^1^

Strategy	Addresses	Rationale/explanation	Potential limitation
Pleiotropy analyses	Genetic confounding	Test association between instrument(s) and wide range of potential confounders	Does not test for association with unknown confounders
Exclusion of nonspecific SNPs	Genetic confounding	SNPs associated with multiple exposures may introduce pleiotropy	Power may be limited to detect nonspecific associations; exclusion of nonspecific SNPs can also introduce bias into the analysis
Weighted median estimator	Violation of all MR assumptions	Sensitivity analysis allowing 50% of the instruments to be invalid	At least 50% of the genetic proxies must be valid instruments
MR-Egger regression	Direct effects/horizontal pleiotropy	Sensitivity analysis allowing all instruments to be subject to direct effects (i.e., horizontal pleiotropy)	The InSIDE assumption is required: strength of the gene-exposure association must not correlate with the strength of bias due to horizontal pleiotropy
Gene-environment interactions	Genetic confounding	Association should only be observed in certain exposure subgroups (e.g., smoking instruments in ever- compared with never-smokers)	Limited number of available gene-environment interactions; can introduce collider bias
Multiple independent instruments	Genetic confounding	Association across multiple independent genomic regions should be robust to confounding	Power likely to be limited for individual genetic variants
Two-sample approaches	Weak instrument bias and low power	Allows larger sample sizes because measurement of the exposure is not required in all samples; bias from weak instruments is toward the null, rather than the confounded observational association	Samples must be independent and representative of the same population; less flexible than 2SLS
Multi-SNP instruments	Weak instrument bias and low power	Instrument will explain more of the variance in the exposure, reducing impact of weak instruments bias and increasing power	Requires multiple GWAS significant hits; increases chance of pleiotropy
External weights for 2SLS	Weak instrument bias	Weight the first stage by SNP-exposure effect from an external study	Precisely estimated external weights must be unavailable

1 GWAS, genome-wide association study; InSIDE, Instrument Strength Independent of Direct Effect; MR, Mendelian randomization; SNP, single nucleotide polymorphism; 2SLS, 2-stage least squares.

### Assessment of association with measured confounders

In an MR analysis, the association between the genetic instrument or instruments and a wide range of potential confounders can be assessed. The MR assumptions can be viewed as plausible when the number of associations is no greater than expected by chance, as is generally observed empirically (70). An exclusive association with the target exposure provides further evidence supporting the MR assumptions. For example, in an MR study of CRP and CAD, the genetic instrument for CRP was associated with CRP concentrations but was not associated with a wide range of other vascular risk factors (13). However, a limitation is that associations can only be tested for measured traits and residual confounding by unknown factors remains a possibility.

### Exclusion of nonspecific genetic instruments

Nonspecific genetic instruments (e.g., SNPs associated with nontarget exposures as well as the target exposure) can be excluded in a sensitivity analysis (18). If a genetic instrument remains associated with an outcome of interest after excluding nonspecific SNPs, evidence for causality may be strengthened. On the other hand, this selection strategy could also introduce bias into an MR study—for example, if the multiple exposures were on the same causal pathway from SNP to disease. In addition, deciding that an SNP is “specific” for an exposure of interest may reflect a lack of statistical power to detect associations with nontarget exposures rather than true biological specificity.

### Detecting and correcting for pleiotropy by statistical and graphical tests

The use of multiple genetic polymorphisms as instruments makes it easier to detect evidence of pleiotropy by statistical and graphical tests. If the estimate of the causal effect is of a consistent magnitude across multiple independent instruments, then pleiotropy is considerably less likely to account for the results, as is observed in MR studies of LDL cholesterol (12). On the other hand, if the causal effects are not consistent across independent instruments (e.g., with some genetic instruments showing unexpectedly large or small effects on the outcome, given the magnitude of their exposure effect), this could be indicative of pleiotropy. Formal statistical tests for such heterogeneity include Cochran’s Q statistic and the likelihood ratio test, which test for consistency across causal effects estimated by Wald ratios and likelihood-based methods, respectively (57). When causal effects have been estimated by 2SLS, heterogeneity across instruments can be assessed by the Sargan test (71). Heterogeneous effects can also be visualized by scatterplots of the gene-outcome and gene-exposure associations and forest plots of Wald ratios for each independent genetic instrument.

**FIGURE 6:**
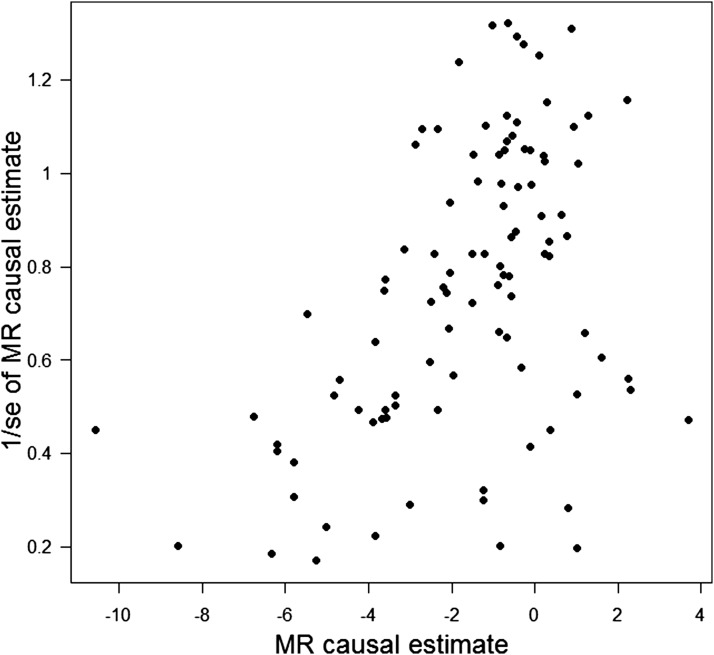
Funnel plot of MR causal estimates against their precision. Each data point corresponds to an individual genetic variant. The *x* axis corresponds to the coefficient of the gene-outcome association divided by the coefficient of the gene-exposure association (i.e., Wald ratios). The funnel plot asymmetry is due to some genetic variants having unusually strong effects on the outcome given their low precision. This asymmetry is indicative of directional pleiotropy. MR, Mendelian randomization.

**FIGURE 7:**
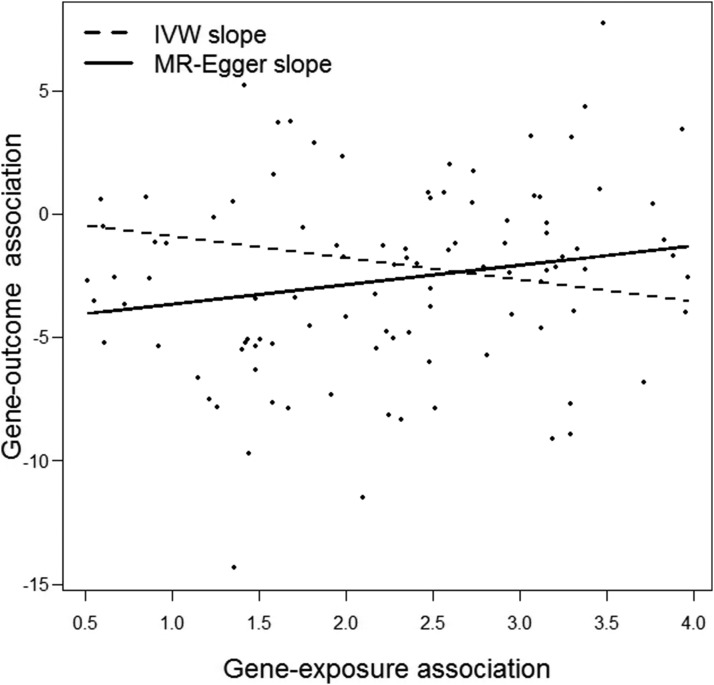
Scatterplot of gene-outcome against gene-exposure associations. Each data point corresponds to an individual genetic variant. The *x* axis corresponds to the coefficient of the gene-exposure association. The *y* axis represents the coefficient of the gene-outcome association. In this example, MR assumption 2 is violated for each genetic variant such that each variant is subject to horizontal pleiotropy or direct effects. As a consequence, the intercept from MR-Egger regression does not pass through zero. The intercept from MR-Egger regression is an estimate of the average direct effect across the genetic variants. The dashed and solid lines correspond to the slopes from IVW and MR-Egger regression, respectively, and can be interpreted as the unit change in the outcome per unit increase in the exposure due to the genetic variants. Unlike IVW regression, the intercept in MR-Egger regression is not constrained to pass through zero. IVW, inverse-variance–weighted; MR, Mendelian randomization.

Pleiotropy is also detectable by asymmetry in a funnel plot, in which the MR estimate is plotted against its precision, and in MR-Egger’s test, a regression of the gene-outcome on the gene-exposure associations with the intercept unconstrained to pass through zero (72) (**Figures 6** and **7**). The intercept from MR-Egger regression provides a formal statistical test for the presence of directional pleiotropy, because when the gene-exposure association is zero the gene-outcome association should also be zero (Figure 7). The contrary implies the presence of a pathway between gene and outcome that is not mediated by the exposure (i.e., a direct effect). The slope from MR-Egger regression corresponds to the exposure-outcome association adjusted for directional pleiotropy. This approach exploits the idea that bias due to directional pleiotropy is analogous to publication bias in a meta-analysis and relaxes the exclusion restriction assumption of MR (assumption 2 discussed above). The method does, however, require other MR assumptions to hold. In particular, the strength of the gene-exposure association should not correlate with the strength of bias due to pleiotropy [the so-called Instrument Strength Independent of Direct Effect (InSIDE) assumption (72)], which could occur if the genetic proxy correlated with confounders of the exposure-outcome association. The InSIDE assumption in MR-Egger may also be violated in a case-control setting, because outcome-dependent sampling can induce associations between genetic instruments and confounders in the case-control sample. However, it is unlikely that this would substantially affect type I error rates, particularly if the disease is rare. An important additional consideration is that MR-Egger can only detect evidence of directional pleiotropy. In other words, the effect on the outcome due to pleiotropy should be in the same direction for each genetic instrument, consistently biasing the gene-outcome association upward or downward. Pleiotropic effects in opposing directions will tend to “balance out” and will therefore be less detectable. An advantage of the approach is that it gives a valid estimate even if all genetic instruments are subject to directional pleiotropy.

MR-Egger regression should be considered as a type of sensitivity analysis rather than as an alternative to the more common approaches described above. Whereas MR-Egger regression relaxes some MR assumptions for all SNPs, other sensitivity analyses allow complete violation of MR assumptions for subsets of SNPs (73–75). For example, a weighted median estimator provides an estimate of the causal effect if at least 50% of the genetic instruments satisfy MR assumptions (75). Thus, if <50% of the genetic instruments are invalid, the weighted median still gives a valid causal estimate. An advantage of the weighted median and MR-Egger approaches is that both can be implemented by using summary data, whereas other similar methods require individual-level data (73, 74). A limitation of MR-Egger is that it tends to suffer from low statistical power for effect estimation.

## INTERPRETATION OF AN MR STUDY

Interpretation of an MR study depends on a number of factors, including a consideration of whether MR assumptions can be considered broadly satisfied. It is also common practice to compare etiologic estimates from observational and MR studies, but such comparisons should be treated with caution because there are many circumstances under which the magnitude of genetic and observational associations could differ. These include reverse causation and confounding in the observational study but also the various assumptions and limitations discussed above. Nevertheless, if an MR study finds strong evidence of association that persists in sensitivity analyses, and if the strategies discussed above indicate no major violations of assumptions, then the association between exposure and outcome can be viewed as compatible with causality.

A particularly promising application of MR is in the prioritization of targets for disease prevention, such as in RCTs. For example, RCTs of proprotein convertase subtilisin/kexin type 9 (PCSK9) inhibitors for reducing LDL cholesterol were designed on the basis of genetic supporting evidence (76, 77). Negative results from MR studies can also help deprioritize interventions, as in the case of CRP and CAD (13, 14). An assumption underlying the interpretation and application of MR is the idea that we can infer causality for other sources of variation (e.g., physiologic, dietary, and therapeutic) on the basis of genetic evidence. Although there are theoretical situations in which this may not apply (such as in the case of canalization), the principle is generally well established in the pharmacologic setting (78). For example, genetic evidence for causation increases 4-fold across the drug discovery pipeline, from the preclinical stage to approved drugs (78). On the basis of this observation, it has been estimated that the prioritizing of genetically supported targets could double the success rate of drug development (78).

## CONCLUSIONS AND FINAL CONSIDERATIONS

In this article we have considered the design, assumptions, and limitations of MR, as well as the various approaches and strategies that can be used to strengthen causal inference. The 2-sample strategy described above should prove particularly useful to epidemiologic studies of nutrition. In one recent GWAS (79), potential genetic instruments for a wide range of metabolites relevant to nutrition were uncovered, including biomarkers of cofactors and vitamins, carbohydrates, amino acids, xenobiotics, peptides, and lipids. In principle, genetic instruments for biomarkers of nutrition defined in one study could be investigated in large, easily accessible case-control collections of noncommunicable diseases, such as the CARDIoGRAM consortium of CAD, by using only summary data (Table 3). The findings from such studies could subsequently be used to prioritize promising biomarkers for investigation in follow-up observational or intervention studies or used to strengthen the evidence base for public health policies relating to diet. Given the growing availability of open-access data from large GWAS consortia and population biobanks, the feasibility of MR studies of modifiable exposures is likely to increase markedly in the near future.

## Footnotes

5 Abbreviations used: *ADH1B*, alcohol dehydrogenase 1B; *ALDH2*, aldehyde dehydrogenase 2; *apo(a)*, apolipoprotein(a); CAD, coronary artery disease;* CHRNA5-A3-B4*, cholinergic receptor nicotinic α 5 subunit - α 3 subunit - β 4 subunit (nicotinic receptor gene cluster); CRP, C-reactive protein; EC-SOD, extracellular superoxide dismutase; *FTO*, fat mass and obesity associated; GWAS, genome-wide association study; IL-6R, IL-6 receptor; Lp(a), lipoprotein(a);* LPA*, apolipoprotein(a) gene; MR, Mendelian randomization; PCSK9, proprotein convertase subtilisin/kexin type 9; RCT, randomized controlled trial; SNP, single nucleotide polymorphism; *SOD3*, superoxide dismutase 3; 2SLS, 2-stage least-squares.
